# The Recent 2023 Earthquake in Nepal: A Global Health Perspective

**DOI:** 10.31729/jnma.8642

**Published:** 2024-07-31

**Authors:** Bibek Giri, Ashesh Malla, Vijay Kumar Chattu

**Affiliations:** 1 Research Institute for Collaborative Development, Kathmandu, Nepal; 2 Department of Community Medicine and Public Health, Chitwan Medical College, Tribhuvan University, Chitwan, Nepal; 3 Department of Occupational Science & Occupational Therapy, Temerty Faculty of Medicine, University of Toronto, Toronto, Canada

**Keywords:** *community engagement*, *earthquake*, *healthcare delivery*, *mitigation*, *natural disasters*

## Abstract

As a seismic hotspot, Nepal has endured many catastrophic earthquakes, including the 2023 Jajarkot quake. These quakes worsen the existing fragilities, resulting in difficulties in accessing healthcare, outbreaks of infectious diseases, mental health problems, and nutritional shortfalls. The article examines the complex web of health consequences, such as infectious and non-infectious diseases and malnutrition, highlighting the need for a global health lens in tackling these issues. It also reveals the long-term health effects, such as mental health disorders and increased disease susceptibility, that emerge after the quake and the importance of enhancing coordination and communication, enforcing building codes, and assisting affected communities in response to the seismic hazards. It discusses the role of climate change in seismic risks and the need for research, innovation, and adaptability in global health interventions, suggesting measures such as strengthening primary healthcare, preventing avoidable health problems through education, and improving supply chains. The article calls for a holistic approach to building resilient health systems, emphasizing community engagement, prevention, and preparedness to protect the health of vulnerable groups in seismic regions.

## INTRODUCTION

Nepal's history is marked by several tragic earthquakes, such as the 1934 Bihar-Nepal earthquake, 2015 Gorkha earthquake, and recent 2023 Jajarkot earthquake. A tremor of 6.4 shook the land of Nepal on the 4th of November, 2023. When the clock struck 12, the epicenter was near Jajarkot and Rukum West, where at least 129 deaths and 140 were injured.^1,2^ The 2015 earthquake led to loss of nearly 9,000 lives and severe damage of more than 500,000 residences.^2^ The country's exposure to earthquakes is aggravated by rapid urbanization, poor building quality, lack of resource mapping, low resources and, mobilization for response. Thus, Nepal faces challenges and risks in dealing with seismic hazards and ensuring the safety and well-being of its population.^2, 3^

## HEALTH IMPLICATIONS AND CHALLENGES

Although the impact of earthquakes on public health is not limited to the immediate aftermath of a disaster, it can still have medium- and long-term effects on the health and well-being of affected populations. The earthquake can bring more woes than just the shake as it can make people sick, both in body and mind, by hurting and impairing them with diseases and disabilities.^4^ The quake can also hamper the healthcare system, making it difficult to reach and help those in need. Moreover, it can worsen the existing gaps and vulnerabilities by making the health situation dire.^5^ Examining the event from a global health perspective is crucial, considering the various factors influencing health, such as social, environmental, economic, and political conditions.

Moreover, it recognizes the complex and dynamic relationships among health systems and actors worldwide, which can affect the response and recovery efforts. By adopting a global health perspective, we can better understand the needs and priorities of people affected by the earthquake and mobilize resources and coordination to address them effectively. We can also monitorand evaluate the health outcomes and impacts of the earthquake and learn from the experience to enhance resilience and preparedness for future disasters.^6^ The earthquake also increased the risk of various diseases, such as respiratory infections, diarrheal diseases, and vector-borne diseases, due to overcrowding, poor sanitation, lack of safe water, and disruption of health services.^7^ Moreover, the earthquake increased the risk of non-communicable diseases, such as cardiovascular diseases, diabetes, and mental health disorders, due to stress, lack of access to medicines and care, and loss of livelihoods and social support.^7,8^ Furthermore, the earthquake increased the risk of malnutrition, especially among children, pregnant and lactating women, and the elderly, due to food insecurity, disruption of feeding practices, and lack of micronutrient supplementation.^9^ The earthquake in Nepal posed several challenges for the local healthcare infrastructure, affecting health response and recovery. According to the Government of Nepal, the earthquake damaged or destroyed many health facilities and houses in the affected area. This limits the availability and quality of health services. The earthquake also injured or displaced many health workers, who had to attend their families and homes, affecting the continuum of healthcare delivery. The earthquake also damaged roads, bridges, and communication networks and hampered access to and provision of emergency and specialized care for the people. The earthquake also overwhelmed the existing health systems. It requires the involvement of multiple sectors, such as the Government of Nepal, national and international relief agencies, and private sector providers, leading to gaps, overlaps, and inefficiencies in health response and coordination.^10^

## LONG TERM HEALTH IMPACT

Nepal's earthquake was a catastrophic event that had serious repercussions on the health and well-being. Among the potential long-term health impacts, mental health problems were prevalent, such as earthquake-induced trauma, stress, anxiety, depression, and posttraumatic stress disorder (PTSD), especially for those who lost their loved ones, homes, and livelihoods. These psychological effects may last for years and impair the quality of life, social functioning, and coping skills of affected individuals, who may face inadequate and inaccessible mental health services, particularly in rural areas.^11^ Moreover, the earthquake increases the risk of infectious diseases, as it damages the water and sanitation infrastructure, leading to unsafe drinking water and poor hygiene. This disaster facilitated the spread of waterborne diseases, such as cholera, typhoid, dysentery, and hepatitis. The earthquake also hampered immunization programs and the supply of essential medicines, which made the population more vulnerable to vaccine-preventable diseases such as measles, tetanus, and diphtheria.^12^

Furthermore, overcrowding and poor living conditions in temporary shelters have enabled the transmission of respiratory infections, such as tuberculosis, pneumonia, and influenza. Additionally, the earthquake may have deteriorated the health outcomes of people with chronic diseases such as diabetes, hypertension, cardiovascular diseases, and cancer, as it disrupts the continuity of care, availability of medications, and access to health facilities for these patients. The earthquake may also have influenced the risk factors for chronic diseases, such as smoking, alcohol consumption, physical inactivity, and unhealthy diet.^12^ Finally, the earthquake caused food insecurity and malnutrition, destroying many crops, livestock, and food stocks, affecting food production and income. It also disturbs food distribution and markets, causing food shortages and price hikes. These effects have implications for the health and livelihood of Nepalis, as food insecurity increases the risk of malnutrition, especially among children, pregnant women, and lactating mothers who have higher nutritional needs. Malnutrition may compromise the affected individuals' growth, development, immunity, and cognitive function and increase their susceptibility to infections and chronic diseases.^13,14^ Amidst the seismic upheaval, Nepal's trembling earthquake fractured buildings and unleashed invisible threats that ripple through communities. Within the ominous spectrum of disease outbreaks there are many challenges. Cholera and dysentery, akin to vengeful spirits, haunt vulnerable souls in the aftermath of the Nepal earthquake, fueled by a lack of shelter, contaminated water, and poor sanitation. Tuberculosis, a silent assailant, thrives and may stealthily advance through coughs and sputum, claiming lives beyond the tremors. The shadowy nemesis, scrub typhus, and lurks in the underbrush weave its relentless grip across humans, animals, and chigger mites, as recent fatal outbreaks attest. Survivors cling to life in makeshift camps, but clean water and toilets remain scarce, leaving them vulnerable to unseen foes. Diseases, akin to relentless aftershocks, continue to threaten their fragile existence.^15,16^

## STRENGTHENING COORDINATION AND COMMUNICATION EFFORTS

The Nepal earthquake exposed the vulnerability of numerous structures and infrastructure that did not adhere to seismic codes and standards. It is crucial to enforce building regulations while retrofitting or reconstructing existing unsafe buildings to mitigate future risks. The earthquake significantly affected livelihoods, especially in rural areas, where agriculture is the primary source of income. Supporting affected communities through cash transfers, agricultural assistance, skills training, and improved market access is essential for recovery and resilience. Nepal's disaster management system has revealed deficiencies in coordination, communication, and resource allocation. Strengthening institutional frameworks, enhancing stakeholder capacity, and promoting community awareness are vital to disaster preparedness. Local and national leadership played a crucial role in response and recovery, emphasizing the need to respect their decisions, provide resources, and build long-term capacities. Regional and international collaboration is imperative, given the seismic risks of the Himalayan region. Sharing data, best practices, and policy cooperation can enhance disaster risk reduction efforts.^17, 18^

## ROLE OF MITIGATION AND COMMUNITY ENGAGEMENT:

Nepal government are implementing many strategies for the future disaster management. The Health and Emergency Operation Center (HEOC) has been established in 2014 with WHO support, is pivotal in disaster and public health emergency management. Serving as the Ministry of Health and Population (MoHP's) secretariat, it was instrumental during the 2015 earthquake and subsequent crises like floods and the COVID-19 pandemic. The HEOC also enhances preparedness through hospital networks, emergency teams, medical supplies stockpiling, and health system assessments, cluster coordination, bolstering Nepal's health security. In addition, acknowledging the importance of distributed management, all seven provinces of Nepal have set up Provincial Health Emergency Operation Centers (PHEOCs).^19^ In addition, following the destructive 6.4 magnitude earthquake in western Nepal on November 4, 2023, a range of readiness, alleviation, and rehabilitation measures have been launched to aid the impacted communities. The quake, which led to considerable destruction and over 150 fatalities, has elicited widespread domestic and global efforts to meet the urgent and enduring requirements of those affected.

After the earthquake, Nepal government and many international agencies like United Nation (UN) had been mobilized immediately. Nepal Government with the collaboration of Nepalese Army started the search operation for the lost people. Many UN agencies like World health organization (WHO), World Food program (WFP), United Nation International Children Emergency Fund (UNICEF) has played a crucial role in delivering essential supplies such as temporary housing, sustenance, health kits, and various nonfood essentials to those communities impacted by the disaster. Similarly, UNICEF has been instrumental in providing materials like tarpaulins, blankets, sanitation kits, and provisional sanitation facilities to avert the spread of diseases. The WHO has dispatched emergency health kits to cater to the population's urgent health requirements. The initiatives encompass trauma care and medical treatment for individuals harmed by the earthquake. In addition, WFP provided 6 metric tons of food to emergency area where UNICEF provided almost 1067 sets of tarpaulins and 1,300 pieces of blankets to damaged population. Furthermore, UNICEF have provided Hygiene kits, including buckets, mugs, the water purifier 'Piyush', and temporary toilets, have been provided as part of the Water Sanitation and Hygiene supplies for the displaced individuals in the two districts most impacted.^20^

Bolstering global health responses to earthquake disasters requires emphasis on the importance of interconnected strategies. First, early warning systems must be reinforced through investment in seismic monitoring and rapid alert mechanisms. At the same time, public education and awareness campaigns should inform communities about earthquake risks and essential procedures.^21^ Second, healthcare infrastructure should be fortified by retrofitting existing facilities for seismic resilience, maintaining stockpiles of critical medical supplies, and providing disaster response training for healthcare professionals.^21^ Third, community engagement and empowerment can enhance preparedness by involving local communities in risk assessments, implementing community health worker programs, and integrating psychosocial support into disaster response.^22^ To ensure effective coordination and collaboration, inter-agency cooperation is imperative to prevent redundancy, while international collaboration fosters the sharing of best practices and resources. Clear risk communication tailored to local contexts and languages, rigorous data collection, and research initiatives will yield comprehensive insights into immediate needs and long-term impacts.

Moreover, acknowledging the influence of climate change on seismic risks is critical, and resource allocation, including dedicated emergency funds and private sector engagement, is vital.^23^ Lastly, research and innovation, encompassing technological solutions and evidence-based practices, are essential components of a comprehensive approach. It is crucial to recognize that effective global health responses hinge on cooperation, adaptability, and steadfast commitment to safeguard vulnerable populations.^23^

## WAY FORWARD

Improving local healthcare infrastructure and resilience is crucial for guaranteeing an efficient response to disasters and enhancing long-term health outcomes. First, capitalize on current response efforts to bolster overall health system resilience by strengthening pandemic preparedness and prioritizing essential public health functions crucial for comprehensive emergency risk management. Second, it establishes a robust foundation for primary healthcare by reinforcing community-based care and empowering local health workers to provide essential services. Third, this study aimed to prevent avoidable health issues through health promotion and community education to encourage healthier lifestyles and disease prevention. Consider innovative supply chain optimization for medical equipment, medications, and vaccines and explore telemedicine and technology-driven healthcare delivery methods. Finally, enhancing environmental health by improving air quality, sanitation, waste management, and biodiversity conservation, as these factors significantly impact community well-being. Resilient health systems must be developed using a bottom-up approach that emphasizing community engagement, prevention, and preparedness.
